# Caregiving Preparedness and Self‐Efficacy Following Alzheimer's Disease Biomarker Disclosure: Findings from Care Partners of Individuals with Cognitive Impairment

**DOI:** 10.1002/alz70856_104881

**Published:** 2026-01-07

**Authors:** Sara J Feldman, Mary Lesniak, Gloria Whitaker, Haley Kohl, Keiris Surita, J. Scott Roberts, Benjamin M. Hampstead, Annalise Rahman‐Filipiak

**Affiliations:** ^1^ University of Michigan School of Public Health, Ann Arbor, MI, USA; ^2^ Research Program on Cognition & Neuromodulation Based Interventions, University of Michigan, Ann Arbor, MI, USA; ^3^ Research Program on Cognition & Neuromodulation Based Interventions ‐ University of Michigan, Ann Arbor, MI, USA; ^4^ Research Program on Cognition and Neuromodulation Based Interventions, University of Michigan, Ann Arbor, MI, USA; ^5^ University of Michigan, Ann Arbor, MI, USA; ^6^ Research Program on Cognition and Neuromodulation‐Based Interventions, University of Michigan, Ann Arbor, MI, USA

## Abstract

**Background:**

Alzheimer's disease (AD) biomarker disclosure can help personalize care for patients and their care partners. However, little research has focused on the impact of biomarker disclosure on care partners, particularly in relation to caregiving preparedness and self‐efficacy. Understanding how care partners' caregiving preparedness and self‐efficacy can change following biomarker disclosure is crucial for being able to improve care partner support.

**Method:**

71 care partners [60.6% female; 88.7% White; mean age 67.6 ± 9.0 years] enrolled in the study with their respective participant with a diagnosis of Mild Cognitive Impairment, AD dementia or other dementia and underwent positron emission tomography amyloid and tau disclosure. Care partners completed the Preparedness for Caregiving Scale (PCS) and the Revised Scale for Caregiving Self Efficacy (RSCSE‐subscale Controlling Upsetting Thoughts) at 4 timepoints: baseline, immediately post‐disclosure, 1‐week and 6‐week post disclosure. Linear mixed effects multivariable regression analyses were conducted to assess the impact of AD biomarker disclosure.

**Result:**

*PCS*: Care partners reported significantly less caregiving preparedness if the participant had AD dementia (coefficient: ‐0.63, 95% CI ‐1.25, ‐0.01), and the effect was significantly dependent if tau+ was disclosed (coefficient: 0.56, 95% CI 0.06, 1.05). *RSCSE*: Disclosure of tau+ to the participant was significantly associated with increased care partner self‐efficacy (coefficient: 14.86, 95% CI 4.3, 25.4). Disclosure of amyloid+ was not significantly associated with caregiving preparedness or self‐efficacy.

**Conclusion:**

This study underscores the importance of biomarker status when designing and implementing support services for care partners following biomarker disclosure. A likely explanation of caregiving preparedness being lower and self‐efficacy higher when tau+ was disclosed – but the finding being null for amyloid+ – is that care partners of persons with cognitive impairment likely have experience at managing the emotional toll of caregiving but may not be prepared for thinking about the long‐term prospect of caregiving following a disclosed tau+ result, an indicator of dementia severity. As the caregiver's role becomes more complex following information about what potentially to expect in the patient (e.g., disease progression), it is crucial to design and implement tailored support services for care partners following biomarker disclosure.

